# Poly[(μ_2_-quinoline-3-carboxyl­ato-κ^2^ *N*:*O*)(μ_2_-quinoline-3-carboxyl­ato-κ^3^ *N*:*O*,*O*′)cadmium]

**DOI:** 10.1107/S1600536812001031

**Published:** 2012-01-18

**Authors:** Xing Wang, Yong-Sheng Yan, He-Yi Sun, Shen-Tang Wang, Chun-Bo Liu

**Affiliations:** a School of Chemistry and Chemical Engineering, Jiangsu University, Zhenjiang 212013, People’s Republic of China

## Abstract

In the title compound, [Cd(C_10_H_6_NO_2_)_2_]_*n*_, the Cd^II^ atom is coordinated by three O atoms and two N atoms from four quinoline-3-carboxyl­ate (*L* ^−^) ligands, leading to a distorted trigonal–bipyramidal geometry. The *L* ^−^ ligands link the Cd^II^ atoms into a plane parallel to (100), with one ligand being tridentate, coordinating *via* the N atom and chelating a second Cd atom, and the other being bidentate, bridging two Cd atoms *via* the N and one O atom.. This two-dimensional network extends into a double-layer network by π–π inter­actions, with centroid–centroid distances of 3.680 (2) and 3.752 (2) Å. Another type of π–π inter­action between pyridine rings [centroid–centroid distance = 3.527 (2) Å] leads to a three-dimensional supra­molecular architecture.

## Related literature

For background to the applications of cadmium coordination polymers and nicotinic acids, see: Niu *et al.* (2006 ▶); Song *et al.* (2006 ▶), Chen (2003) ▶; Chi *et al.* (2007 ▶); Lu *et al.* (2007 ▶). For a closely related structure, see: Hu *et al.* (2007 ▶).
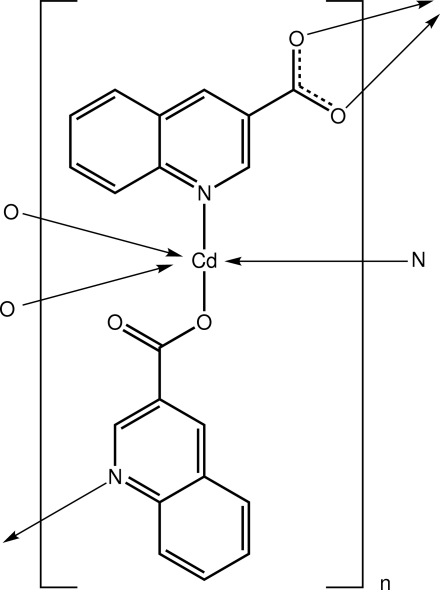


## Experimental

### 

#### Crystal data

[Cd(C_10_H_6_NO_2_)_2_]
*M* *_r_* = 456.72
Monoclinic, 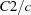

*a* = 28.5458 (19) Å
*b* = 8.2274 (5) Å
*c* = 15.381 (1) Å
β = 112.708 (1)°
*V* = 3332.3 (4) Å^3^
*Z* = 8
Mo *K*α radiation
μ = 1.34 mm^−1^
*T* = 153 K
0.13 × 0.11 × 0.10 mm

#### Data collection

Rigaku Saturn 724+ CCD area-detector diffractometer
Absorption correction: multi-scan (*CrystalClear*; Rigaku, 2007 ▶) *T*_min_ = 0.845, *T*_max_ = 0.910
7361 measured reflections
3014 independent reflections
2659 reflections with *I* > 2σ(*I*)
*R*_int_ = 0.015

#### Refinement

*R*[*F* ^2^ > 2σ(*F* ^2^)] = 0.019
*wR*(*F* ^2^) = 0.051
*S* = 1.10
3014 reflections
244 parameters
H-atom parameters constrained
Δρ_max_ = 0.33 e Å^−3^
Δρ_min_ = −0.25 e Å^−3^

### 

Data collection: *CrystalClear* (Rigaku, 2007 ▶); cell refinement: *CrystalClear*; data reduction: *CrystalClear*; program(s) used to solve structure: *SHELXS97* (Sheldrick, 2008 ▶); program(s) used to refine structure: *SHELXL97* (Sheldrick, 2008 ▶); molecular graphics: *CrystalClear* (Rigaku, 2007 ▶) and *DIAMOND* (Brandenburg, 1998 ▶); software used to prepare material for publication: *SHELXTL* (Sheldrick, 2008 ▶).

## Supplementary Material

Crystal structure: contains datablock(s) global, I. DOI: 10.1107/S1600536812001031/vn2029sup1.cif

Structure factors: contains datablock(s) I. DOI: 10.1107/S1600536812001031/vn2029Isup2.hkl

Additional supplementary materials:  crystallographic information; 3D view; checkCIF report

## Figures and Tables

**Table 1:** Selected bond lengths (Å)

Cd1—N1^i^	2.3410 (16)
Cd1—N2	2.3188 (17)
Cd1—O1	2.1625 (15)
Cd1—O3^ii^	2.3858 (15)
Cd1—O4^ii^	2.2770 (15)

Symmetry codes: (i) 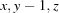; (ii) 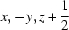.

**Fig. 1.**
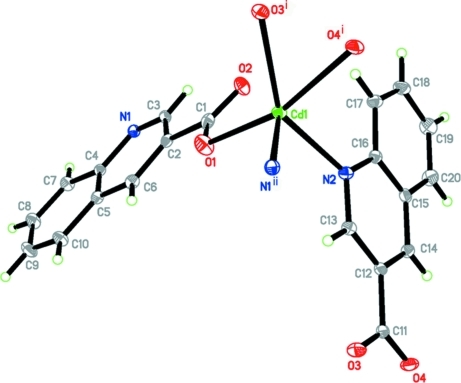
View of the title compound showing displacement ellipsoids (drawn at a 30% probability level) and labeling. H atoms are drawn as a small spheres of arbitrary radius. [symmetry codes: (i) x, - y, z + 1/2; (ii) x, y - 1, z.]

**Fig. 2.**
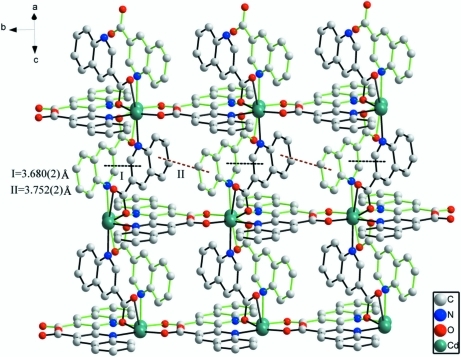
2D double-layer structure and π-π stacking interactions between different 2D layers. All hydrogen atoms were omitted for clarity.

**Fig. 3.**
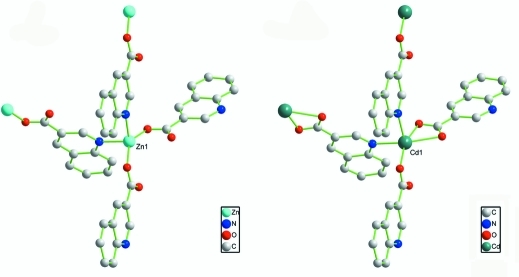
The comparision of the metal coordination environment between a reported isostructural Zn analogue and the title compound.

## supplementary crystallographic information

### Comment

To date, much effort has been made on the construction of cadmium coordination polymers with a wide variety of topological structures which may possess promising perspectives toward molecular luminescent materials (Chi *et al.*, 2007; Niu *et al.*, 2006; Song *et al.*, 2006; Lu *et al.*, 2007). It is well known that nicotinic acid has been proved to be effective for constructing coordination polymers due to the versatile coordination fashion (Chen *et al.*, 2003; Song *et al.*, 2006). Compared with nicotinic acid, the structurally similar quinoline-3-carboxylic acid (H*L*) have been chosen to construct a new coordination polymer. Here, we report on the crystal structure of the title compound.

There is one cadmium (II) atom and two independent *L*^-^ ligands in the asymmetric unit. The Cd (II) atom is five-coordinated by two N atoms [Cd1—N1^ii^=2.341 (2) Å, Cd1—N2=2.319 (2) Å] and three O atoms [Cd1—O1=2.163 (2) Å, Cd—O3^i^=2.386 (2) Å, Cd—O4^i^=2.277 (2) Å] from four *L*^-^ ligands, showing a distorted trigonal bipyramidal coordination geometry (Fig. 1). The *L*^-^ ligand containing the N1 atom, acts as bis-monodentate mode toward cadmium centers with pyridine nitrogen atoms linking the cadmium atom and the carboxylate group linking the cadmium atom in a monodentate fashion, leading to the formation of a 1D chain structure along the the *b* axis. The 1D chains are linked into a 2D layer network by bis-chelating *L*^-^ ligand containing the N2 atom, There is in addition a 2D double-layer structure (black bond and green bond) which is connected by π–π interactions with the centroid to centroid distances of 3.680 (2) and 3.752 (2) Å, respectively (Fig. 2). The 2D double-layers are parallel to the (100) plane, and linked to each other by another type of π–π interaction between pyridine rings [centroid-to-centroid 3.527 (2) Å], resulting in a 3D supramolecular architecture.

There is a reported isostructural Zn analogue (Hu *et al.*, 2007) which has a tetrahedral environment with *L*^-^ in a bis-monodentate mode, while the title compound shows a distorted trigonal bipyramidal coordination geometry with *L*^-^ in bis-monodentate and bis-chelating modes, respectively (Fig. 3). This comparison reveals the influence of different metal ion on the coordination mode of the ligand.

### Experimental

Quinoline-3-carboxylic acid (H*L*) was purchased commercially and used without further purification. A mixture of CdCl_2_ (18.400 mg, 0.1 mmol), and H*L* (17.300 mg, 0.1 mmol) was dissolved in a 10 mL of water with a pH = 6. The resulting mixture was heated in a 15 mL autoclave with Teflon-liner at 438 K for three days. Then the autoclave was slowly cooled to room temperature, and colourless block-shaped crystals were obtained with a yield of 50 %.

### Refinement

All non-hydrogen atoms were refined anisotropically, and hydrogen atoms were positioned geometrically and refined using a riding model with C—H distances of 0.93 Å and *U*_iso_(H)=1.2*U*_eq_(C).

### Crystal data

**Table:**

[Cd(C_10_H_6_NO_2_)_2_]	*F*(000) = 1808
*M**_r_* = 456.72	*D*_x_ = 1.821 Mg m^−^^3^
Monoclinic, *C*2/*c*	Mo *K*α radiation, λ = 0.71073 Å
Hall symbol: -C 2yc	Cell parameters from 6971 reflections
*a* = 28.5458 (19) Å	θ = 2.8–29.2°
*b* = 8.2274 (5) Å	µ = 1.34 mm^−^^1^
*c* = 15.381 (1) Å	*T* = 153 K
β = 112.708 (1)°	Prism, colourless
*V* = 3332.3 (4) Å^3^	0.13 × 0.11 × 0.10 mm
*Z* = 8	

### Data collection

**Table:**

Rigaku Saturn 724+ CCD area-detector diffractometer	3014 independent reflections
Radiation source: fine-focus sealed tube	2659 reflections with *I* > 2σ(*I*)
graphite	*R*_int_ = 0.015
Detector resolution: 28.5714 pixels mm^-1^	θ_max_ = 25.3°, θ_min_ = 3.1°
ω scans	*h* = −34→34
Absorption correction: multi-scan (*CrystalClear*; Rigaku, 2007)	*k* = −9→9
*T*_min_ = 0.845, *T*_max_ = 0.910	*l* = −12→18
7361 measured reflections	

### Refinement

**Table:**

Refinement on *F*^2^	Primary atom site location: structure-invariant direct methods
Least-squares matrix: full	Secondary atom site location: difference Fourier map
*R*[*F*^2^ > 2σ(*F*^2^)] = 0.019	Hydrogen site location: inferred from neighbouring sites
*wR*(*F*^2^) = 0.051	H-atom parameters constrained
*S* = 1.10	*w* = 1/[σ^2^(*F*_o_^2^) + (0.0291*P*)^2^ + 0.9823*P*] where *P* = (*F*_o_^2^ + 2*F*_c_^2^)/3
3014 reflections	(Δ/σ)_max_ = 0.002
244 parameters	Δρ_max_ = 0.33 e Å^−^^3^
0 restraints	Δρ_min_ = −0.25 e Å^−^^3^

### Special details

**Table:**

Geometry. All esds (except the esd in the dihedral angle between two l.s. planes) are estimated using the full covariance matrix. The cell esds are taken into account individually in the estimation of esds in distances, angles and torsion angles; correlations between esds in cell parameters are only used when they are defined by crystal symmetry. An approximate (isotropic) treatment of cell esds is used for estimating esds involving l.s. planes.
Refinement. Refinement of F^2^ against ALL reflections. The weighted R-factor wR and goodness of fit S are based on F^2^, conventional R-factors R are based on F, with F set to zero for negative F^2^. The threshold expression of F^2^ > 2sigma(F^2^) is used only for calculating R-factors(gt) etc. and is not relevant to the choice of reflections for refinement. R-factors based on F^2^ are statistically about twice as large as those based on F, and R- factors based on ALL data will be even larger.

### Fractional atomic coordinates and isotropic or equivalent isotropic displacement parameters (Å^2^)

**Table:**

	*x*	*y*	*z*	*U*_iso_*/*U*_eq_	
C1	0.34654 (8)	0.4503 (3)	0.04346 (16)	0.0215 (5)	
C2	0.32133 (8)	0.6060 (2)	−0.00212 (15)	0.0175 (4)	
C3	0.34221 (7)	0.7571 (2)	0.03538 (14)	0.0179 (4)	
H3	0.3719	0.7577	0.0895	0.021*	
C4	0.27770 (8)	0.8986 (2)	−0.08096 (14)	0.0171 (4)	
C5	0.25472 (8)	0.7512 (2)	−0.12288 (15)	0.0184 (4)	
C6	0.27767 (8)	0.6043 (2)	−0.08109 (15)	0.0196 (5)	
H6	0.2630	0.5057	−0.1074	0.023*	
C7	0.25462 (8)	1.0467 (3)	−0.12039 (16)	0.0229 (5)	
H7	0.2697	1.1447	−0.0939	0.028*	
C8	0.20978 (9)	1.0464 (3)	−0.19802 (16)	0.0285 (5)	
H8	0.1940	1.1444	−0.2227	0.034*	
C9	0.18757 (9)	0.9000 (3)	−0.24036 (17)	0.0305 (6)	
H9	0.1577	0.9019	−0.2941	0.037*	
C10	0.20908 (8)	0.7552 (3)	−0.20397 (16)	0.0251 (5)	
H10	0.1938	0.6587	−0.2324	0.030*	
C11	0.40746 (8)	0.0031 (2)	−0.24972 (14)	0.0197 (5)	
C12	0.43039 (8)	0.0798 (2)	−0.15411 (14)	0.0176 (4)	
C13	0.40592 (8)	0.0688 (2)	−0.09095 (14)	0.0186 (4)	
H13	0.3751	0.0139	−0.1106	0.022*	
C14	0.47532 (8)	0.1623 (3)	−0.12613 (15)	0.0203 (5)	
H14	0.4921	0.1728	−0.1671	0.024*	
C15	0.49631 (8)	0.2315 (2)	−0.03555 (14)	0.0191 (5)	
C16	0.46983 (8)	0.2114 (2)	0.02540 (14)	0.0175 (4)	
C17	0.49105 (8)	0.2735 (2)	0.11748 (15)	0.0223 (5)	
H17	0.4741	0.2599	0.1579	0.027*	
C18	0.53653 (9)	0.3542 (3)	0.14796 (16)	0.0273 (5)	
H18	0.5507	0.3935	0.2094	0.033*	
C19	0.56197 (9)	0.3778 (3)	0.08655 (18)	0.0305 (6)	
H19	0.5925	0.4344	0.1074	0.037*	
C20	0.54238 (8)	0.3188 (3)	−0.00257 (17)	0.0275 (5)	
H20	0.5595	0.3361	−0.0424	0.033*	
N1	0.32245 (6)	0.89871 (19)	−0.00119 (12)	0.0168 (4)	
N2	0.42383 (6)	0.13141 (19)	−0.00532 (12)	0.0174 (4)	
O1	0.32218 (6)	0.32240 (18)	0.00681 (12)	0.0311 (4)	
O2	0.38802 (6)	0.45193 (19)	0.11082 (12)	0.0291 (4)	
O3	0.36675 (6)	−0.07521 (18)	−0.27266 (10)	0.0245 (3)	
O4	0.43035 (6)	0.02228 (19)	−0.30471 (10)	0.0268 (4)	
Cd1	0.371129 (5)	0.122196 (17)	0.077597 (10)	0.01700 (7)	

### Atomic displacement parameters (Å^2^)

**Table:**

	*U*^11^	*U*^22^	*U*^33^	*U*^12^	*U*^13^	*U*^23^
C1	0.0258 (12)	0.0175 (11)	0.0264 (12)	0.0042 (10)	0.0158 (10)	0.0032 (9)
C2	0.0195 (11)	0.0155 (10)	0.0209 (11)	0.0021 (9)	0.0117 (9)	0.0024 (8)
C3	0.0165 (11)	0.0186 (11)	0.0186 (11)	0.0012 (9)	0.0068 (9)	0.0008 (9)
C4	0.0174 (10)	0.0187 (11)	0.0170 (11)	0.0003 (9)	0.0086 (9)	0.0014 (8)
C5	0.0182 (10)	0.0185 (10)	0.0200 (11)	0.0000 (9)	0.0091 (9)	0.0000 (9)
C6	0.0230 (11)	0.0142 (10)	0.0232 (12)	−0.0025 (9)	0.0108 (10)	−0.0027 (8)
C7	0.0253 (12)	0.0172 (11)	0.0256 (12)	0.0001 (10)	0.0092 (10)	0.0016 (9)
C8	0.0295 (13)	0.0254 (12)	0.0281 (13)	0.0087 (11)	0.0084 (11)	0.0100 (10)
C9	0.0237 (12)	0.0371 (14)	0.0228 (13)	0.0031 (11)	0.0003 (10)	0.0048 (10)
C10	0.0222 (12)	0.0255 (12)	0.0233 (12)	−0.0031 (10)	0.0040 (10)	−0.0016 (10)
C11	0.0226 (12)	0.0175 (10)	0.0163 (11)	0.0082 (10)	0.0044 (9)	0.0018 (8)
C12	0.0197 (11)	0.0166 (10)	0.0143 (11)	0.0043 (9)	0.0042 (9)	−0.0002 (8)
C13	0.0194 (11)	0.0176 (10)	0.0178 (11)	−0.0007 (9)	0.0060 (9)	0.0002 (8)
C14	0.0208 (11)	0.0224 (11)	0.0188 (11)	0.0031 (9)	0.0090 (9)	0.0018 (9)
C15	0.0172 (11)	0.0183 (10)	0.0195 (12)	0.0028 (9)	0.0046 (9)	0.0004 (9)
C16	0.0201 (11)	0.0128 (10)	0.0182 (11)	0.0026 (9)	0.0057 (9)	0.0012 (8)
C17	0.0266 (12)	0.0218 (11)	0.0176 (11)	−0.0006 (10)	0.0076 (9)	−0.0023 (9)
C18	0.0303 (13)	0.0255 (12)	0.0196 (12)	−0.0020 (11)	0.0024 (10)	−0.0051 (9)
C19	0.0218 (12)	0.0324 (14)	0.0328 (14)	−0.0109 (11)	0.0058 (10)	−0.0083 (10)
C20	0.0232 (12)	0.0304 (12)	0.0309 (14)	−0.0061 (10)	0.0126 (11)	−0.0029 (10)
N1	0.0178 (9)	0.0149 (9)	0.0176 (9)	0.0008 (7)	0.0067 (7)	0.0006 (7)
N2	0.0206 (9)	0.0171 (9)	0.0145 (9)	−0.0007 (7)	0.0067 (7)	0.0002 (7)
O1	0.0345 (9)	0.0137 (7)	0.0371 (10)	0.0016 (7)	0.0048 (8)	0.0015 (7)
O2	0.0267 (9)	0.0256 (9)	0.0300 (9)	0.0065 (7)	0.0054 (7)	0.0070 (7)
O3	0.0273 (9)	0.0259 (8)	0.0190 (8)	−0.0054 (7)	0.0075 (7)	−0.0051 (6)
O4	0.0263 (8)	0.0382 (9)	0.0167 (8)	−0.0031 (7)	0.0090 (7)	−0.0073 (7)
Cd1	0.02126 (10)	0.01480 (10)	0.01451 (10)	0.00057 (6)	0.00642 (7)	0.00132 (6)

### Geometric parameters (Å, °)

**Table:**

C1—O2	1.236 (3)	C12—C13	1.401 (3)
C1—O1	1.267 (3)	C13—N2	1.320 (3)
C1—C2	1.504 (3)	C13—H13	0.9300
C2—C6	1.364 (3)	C14—C15	1.407 (3)
C2—C3	1.403 (3)	C14—H14	0.9300
C3—N1	1.322 (3)	C15—C20	1.410 (3)
C3—H3	0.9300	C15—C16	1.423 (3)
C4—N1	1.388 (3)	C16—N2	1.379 (3)
C4—C7	1.407 (3)	C16—C17	1.404 (3)
C4—C5	1.411 (3)	C17—C18	1.370 (3)
C5—C6	1.406 (3)	C17—H17	0.9300
C5—C10	1.414 (3)	C18—C19	1.410 (3)
C6—H6	0.9300	C18—H18	0.9300
C7—C8	1.373 (3)	C19—C20	1.355 (3)
C7—H7	0.9300	C19—H19	0.9300
C8—C9	1.399 (3)	C20—H20	0.9300
C8—H8	0.9300	N1—Cd1^ii^	2.3410 (16)
C9—C10	1.358 (3)	O3—Cd1^i^	2.3858 (15)
C9—H9	0.9300	O4—Cd1^i^	2.2770 (15)
C10—H10	0.9300	Cd1—N1^iii^	2.3410 (16)
C11—O3	1.255 (3)	Cd1—N2	2.3188 (17)
C11—O4	1.262 (2)	Cd1—O1	2.1625 (15)
C11—C12	1.499 (3)	Cd1—O3^iv^	2.3858 (15)
C11—Cd1^i^	2.658 (2)	Cd1—O4^iv^	2.2770 (15)
C12—C14	1.366 (3)	Cd1—C11^iv^	2.658 (2)
			
O2—C1—O1	124.4 (2)	C15—C14—H14	119.9
O2—C1—C2	120.84 (19)	C14—C15—C20	123.0 (2)
O1—C1—C2	114.72 (19)	C14—C15—C16	118.29 (19)
C6—C2—C3	118.19 (18)	C20—C15—C16	118.76 (19)
C6—C2—C1	120.99 (19)	N2—C16—C17	120.15 (18)
C3—C2—C1	120.81 (19)	N2—C16—C15	120.50 (18)
N1—C3—C2	124.21 (19)	C17—C16—C15	119.35 (19)
N1—C3—H3	117.9	C18—C17—C16	120.3 (2)
C2—C3—H3	117.9	C18—C17—H17	119.9
N1—C4—C7	119.89 (18)	C16—C17—H17	119.9
N1—C4—C5	120.76 (18)	C17—C18—C19	120.2 (2)
C7—C4—C5	119.35 (19)	C17—C18—H18	119.9
C6—C5—C4	118.53 (19)	C19—C18—H18	119.9
C6—C5—C10	122.08 (19)	C20—C19—C18	120.7 (2)
C4—C5—C10	119.38 (19)	C20—C19—H19	119.6
C2—C6—C5	120.16 (19)	C18—C19—H19	119.6
C2—C6—H6	119.9	C19—C20—C15	120.7 (2)
C5—C6—H6	119.9	C19—C20—H20	119.7
C8—C7—C4	119.8 (2)	C15—C20—H20	119.7
C8—C7—H7	120.1	C3—N1—C4	118.13 (17)
C4—C7—H7	120.1	C3—N1—Cd1^ii^	113.66 (13)
C7—C8—C9	120.6 (2)	C4—N1—Cd1^ii^	128.17 (13)
C7—C8—H8	119.7	C13—N2—C16	118.63 (17)
C9—C8—H8	119.7	C13—N2—Cd1	116.90 (14)
C10—C9—C8	120.8 (2)	C16—N2—Cd1	124.15 (13)
C10—C9—H9	119.6	C1—O1—Cd1	105.75 (14)
C8—C9—H9	119.6	C11—O3—Cd1^i^	88.09 (12)
C9—C10—C5	120.0 (2)	C11—O4—Cd1^i^	92.87 (13)
C9—C10—H10	120.0	O1—Cd1—O4^iv^	159.22 (6)
C5—C10—H10	120.0	O1—Cd1—N2	97.38 (6)
O3—C11—O4	122.58 (19)	O4^iv^—Cd1—N2	90.81 (5)
O3—C11—C12	119.90 (19)	O1—Cd1—N1^iii^	101.45 (6)
O4—C11—C12	117.52 (19)	O4^iv^—Cd1—N1^iii^	96.41 (6)
O3—C11—Cd1^i^	63.76 (11)	N2—Cd1—N1^iii^	97.04 (6)
O4—C11—Cd1^i^	58.81 (11)	O1—Cd1—O3^iv^	110.18 (6)
C12—C11—Cd1^i^	176.28 (16)	O4^iv^—Cd1—O3^iv^	56.46 (5)
C14—C12—C13	118.20 (19)	N2—Cd1—O3^iv^	145.40 (6)
C14—C12—C11	121.32 (19)	N1^iii^—Cd1—O3^iv^	97.50 (6)
C13—C12—C11	120.49 (19)	O1—Cd1—C11^iv^	136.51 (7)
N2—C13—C12	124.2 (2)	O4^iv^—Cd1—C11^iv^	28.31 (6)
N2—C13—H13	117.9	N2—Cd1—C11^iv^	118.46 (6)
C12—C13—H13	117.9	N1^iii^—Cd1—C11^iv^	97.88 (6)
C12—C14—C15	120.2 (2)	O3^iv^—Cd1—C11^iv^	28.14 (6)
C12—C14—H14	119.9		
			
O2—C1—C2—C6	174.6 (2)	C17—C18—C19—C20	−1.2 (4)
O1—C1—C2—C6	−4.8 (3)	C18—C19—C20—C15	−0.5 (4)
O2—C1—C2—C3	−5.2 (3)	C14—C15—C20—C19	−177.5 (2)
O1—C1—C2—C3	175.38 (19)	C16—C15—C20—C19	2.2 (3)
C6—C2—C3—N1	−0.4 (3)	C2—C3—N1—C4	1.1 (3)
C1—C2—C3—N1	179.44 (18)	C2—C3—N1—Cd1^ii^	−176.85 (15)
N1—C4—C5—C6	1.1 (3)	C7—C4—N1—C3	178.10 (19)
C7—C4—C5—C6	−178.4 (2)	C5—C4—N1—C3	−1.4 (3)
N1—C4—C5—C10	179.84 (19)	C7—C4—N1—Cd1^ii^	−4.3 (3)
C7—C4—C5—C10	0.3 (3)	C5—C4—N1—Cd1^ii^	176.15 (14)
C3—C2—C6—C5	0.0 (3)	C12—C13—N2—C16	−0.8 (3)
C1—C2—C6—C5	−179.81 (18)	C12—C13—N2—Cd1	172.89 (16)
C4—C5—C6—C2	−0.4 (3)	C17—C16—N2—C13	−177.34 (19)
C10—C5—C6—C2	−179.1 (2)	C15—C16—N2—C13	2.5 (3)
N1—C4—C7—C8	−178.5 (2)	C17—C16—N2—Cd1	9.4 (3)
C5—C4—C7—C8	1.0 (3)	C15—C16—N2—Cd1	−170.71 (14)
C4—C7—C8—C9	−2.1 (4)	O2—C1—O1—Cd1	−5.0 (3)
C7—C8—C9—C10	1.9 (4)	C2—C1—O1—Cd1	174.42 (14)
C8—C9—C10—C5	−0.6 (4)	O4—C11—O3—Cd1^i^	−0.1 (2)
C6—C5—C10—C9	178.2 (2)	C12—C11—O3—Cd1^i^	179.26 (17)
C4—C5—C10—C9	−0.5 (3)	O3—C11—O4—Cd1^i^	0.1 (2)
O3—C11—C12—C14	178.30 (19)	C12—C11—O4—Cd1^i^	−179.27 (15)
O4—C11—C12—C14	−2.3 (3)	C1—O1—Cd1—O4^iv^	33.2 (2)
O3—C11—C12—C13	−1.9 (3)	C1—O1—Cd1—N2	−79.22 (14)
O4—C11—C12—C13	177.47 (19)	C1—O1—Cd1—N1^iii^	−177.98 (13)
C14—C12—C13—N2	−0.9 (3)	C1—O1—Cd1—O3^iv^	79.52 (14)
C11—C12—C13—N2	179.27 (18)	C1—O1—Cd1—C11^iv^	67.38 (17)
C13—C12—C14—C15	1.0 (3)	C13—N2—Cd1—O1	−77.63 (15)
C11—C12—C14—C15	−179.23 (19)	C16—N2—Cd1—O1	95.72 (15)
C12—C14—C15—C20	−179.7 (2)	C13—N2—Cd1—O4^iv^	121.51 (15)
C12—C14—C15—C16	0.7 (3)	C16—N2—Cd1—O4^iv^	−65.14 (15)
C14—C15—C16—N2	−2.5 (3)	C13—N2—Cd1—N1^iii^	24.95 (15)
C20—C15—C16—N2	177.84 (19)	C16—N2—Cd1—N1^iii^	−161.70 (15)
C14—C15—C16—C17	177.42 (19)	C13—N2—Cd1—O3^iv^	139.20 (14)
C20—C15—C16—C17	−2.3 (3)	C16—N2—Cd1—O3^iv^	−47.45 (19)
N2—C16—C17—C18	−179.46 (19)	C13—N2—Cd1—C11^iv^	127.90 (14)
C15—C16—C17—C18	0.7 (3)	C16—N2—Cd1—C11^iv^	−58.75 (16)
C16—C17—C18—C19	1.1 (3)		

Symmetry codes: (i) *x*, −*y*, *z*−1/2; (ii) *x*, *y*+1, *z*; (iii) *x*, *y*−1, *z*; (iv) *x*, −*y*, *z*+1/2.
